# LncRNA HOTAIR Enhances Epithelial-to-mesenchymal Transition to Promote the Migration and Invasion of Liver Cancer by Regulating NUAK1 via Epigenetic Inhibition miR-145-5p Expression

**DOI:** 10.7150/jca.85335

**Published:** 2023-07-24

**Authors:** Dong-xia Chu, Yu Jin, Bing-rong Wang, Yan Jiao, Chao-ke Zhang, Zi-han Guo, Shao-zhuo Hu, Na Li

**Affiliations:** 1 Department of Pathophysiology, College of Basic Medical Sciences, Jilin University, Changchun, Jilin, 130021, P.R. China.; 2 Department of Hepatobiliary and Pancreatic Surgery, The First Hospital of Jilin University, Changchun, Jilin, 130021, P.R. China.

**Keywords:** liver cancer, HOTAIR, miR-145-5p, NUAK1, EMT

## Abstract

LncRNA HOTAIR play important roles in the epigenetic regulation of carcinogenesis and progression in liver cancer. Previous studies suggest that the overexpression of HOTAIR predicts poor prognosis. In this study, through transcriptome sequencing data and *in vitro* experiments, we found that HOTAIR were more highly expressed and there is significantly positive relationship between HOTAIR and NUAK1 in liver cancer tissues and cell lines. miR-145-5p was downregulated and showed negative correlation with HOTAIR and NUAK1. Transfect Sh-HOTAIR, LZRS-HOTAIR, miR-145 mimic, miR-145 inhibitor to change the expression of HOTAIR and miR-145-5p. The addition of HTH-01-015 inhibits the expression of NUAK1. HOTAIR knockdown, miR-145-5p upregulation and NUAK1 inhibition all repressed migration, invasion and metastasis and reversed the epithelial-to-mesenchymal transition in SNU-387 and HepG2 cells. We also showed that HOTAIR recruiting and binding PRC2 (EZH2) epigenetically represses miR-145-5p, which controls the target NUAK1, thus contributing to liver cancer cell-EMT process and accelerating tumor metastasis. Moreover, it is demonstrated that HOTAIR crosstalk with miR-145-5p/NUAK1 during epigenetic regulation. Our findings indicate that HOTAIR/miR-145-5p/NUAK1 axis acts as an EMT regulator and may be candidate prognostic biomarker and targets for new therapies in liver cancer.

## Introduction

Liver cancer is one of the most common malignant tumors that represents a serious threat to human health 1. Liver cancer is highly invasive and shows frequent metastasis, leading to unsatisfactory prognosis for patients 2. Therefore, the discovery of new targets is of great significance for preventing the invasion and metastasis of liver cancer.

The activation of epithelial-to-mesenchymal transition (EMT) mainly refers to the transition from the loss of epithelial cell polarity to the motor mesenchymal phenotype 3, 4. The degradation of the extracellular matrix (ECM) by secretases such as matrix metalloproteinases (MMP) is the main feature of EMT. EMT is also characterized by decreased expression of the epithelial marker E-cadherin and increased expression of mesenchymal markers (N-cadherin, Vimentin) 5. EMT is an important sign of cell invasion and metastasis in liver cancer. Recent studies showed that the lncRNA HOTAIR can affect the expression of E-cadherin, N-cadherin and Vimentin by regulating EMT-related transcription factors and thereby affect the invasion and metastasis of cancer cells 6-9.

Hox transcript antisense intergenic RNA (HOTAIR) is a trans-regulatory long non-coding RNA (lncRNA) located in the Hoxc gene cluster 10. The overexpression of HOTAIR is positively correlated with poor prognosis and tumor recurrence in a variety of cancers 11. The latest research shows HOTAIR knockdown suppressed cell proliferation, migration and invasion, and promoted apoptosis via regulating miR-526b-3p/DHX33 axis in hepatocellular carcinoma (HCC) cells 12. The enhancer of Zeste homolog 2 (EZH2), the catalytic component of histone methyltransferase and PRC2, catalyze the trimethylation of histone H3 in Lys 27 (H3K27me3), thereby regulating gene expression through epigenetic mechanisms 13. Studies had found that HOTAIR could interact with PRC2 to trimethylate H3K27, which could reduce the expression of E-cadherin in gastric cancer 14. HOTAIR enhances EMT by affecting the miR-17-5p/PTEN axis in gastric cancer 15. HOTAIR is highly expressed in HCC, and its high expression enhances the invasion and metastasis of HCC cells 16. However, the mechanism of HOTAIR on the invasion and metastasis of liver cancer needs further study.

NUAK1, also known as ARK5, is a member of the AMPK family. NUAK1 is phosphorylated and activated by AKT 17. NUAK1 has specific roles in cell adhesion, invasion, metastasis, oxidative stress and drug resistance 18-20. In intrahepatic cholangiocarcinoma, NUAK1 regulates EMT and affects invasion and metastasis, while miR-145 and miR-424 can be combined with 3' UTR region to regulate the expression of NUAK1 18, 21. Some studies have reported that the high expression of NUAK1 in ovarian cancer is associated with poor prognosis 22, 23. However, the role of NUAK1 in liver cancer is not yet clear.

MicroRNA (miRNA) play an important role in tumorigenesis and cancer metastasis. For example, miR-145 regulates the proliferation of human breast cancer cells by targeting HBXIP and SOX2, and it can be used as a marker for breast cancer risk assessment 24, 25. miR-145 targets snail1 to increase the radiotherapy sensitivity of colorectal cancer 26. miR-145 increases the sensitivity of esophageal squamous cell carcinoma to cisplatin by directly inhibiting the PI3K/AKT signaling pathway 27. However, the function of miR-145 in liver cancer has not been well investigated.

In this sudy, through transcriptome sequencing data and *in vitro* experiments, we found that HOTAIR were more highly expressed and there is significantly positive relationship between HOTAIR and NUAK1 in liver cancer tissues and cell lines. miR-145-5p was downregulated and showed negative correlation with HOTAIR and NUAK1. Additional experiments revealed that HOTAIR knockdown, miR-145-5p upregulation and NUAK1 inhibition all repressed migration, invasion and metastasis and reversed liver cancer cell-EMT in SNU-387 and HepG2. In addition, HOTAIR also epigenetically downregulates miR-145-5p by binding to PRC2 (EZH2) to activate its target gene NUAK1, thereby promoting EMT in advanced stages of liver cancer. Our findings provide new insights into the mechanisms by which HOTAIR regulate the expression of miR-145-5p/NUAK1.

## Materials and methods

### RNA-sequencing (RNA-seq) and Date acquisition

Total RNA from HepG2 cells transfected with HOTAIR plasmid was isolated by TRIzol extraction, quality-verified by Bionalyzer, and analyzed by transcriptome sequencing (Personalbio, China). The Oligo (dT) magnetic beads are used to enrich the mRNA with polyA structure in the total RNA, and the RNA is interrupted to a fragment of about 300bp in length by means of ion interruption. Isolated RNA was used to prepare cDNA libraries and amplified with primers containing sequences required for Illumina HiSeq sequencing platform. PCR products were cleaned and subjected to 450-bp paired-end sequencing on an Illumina Hi-seq. The RNA-Seq data have been deposited in the NCBI Sequence Read Archive (SRA) with accession number PRJNA758831.

The differentially expressed genes were analyzed by GO enrichment analysis and KEGG pathway analysis. Predict the target gene interacting with NUAK1 through STRING database (https://string-db.org). miR-145-5p with differential expressions in HCC tissues was analyzed in the Cancer Genome Atlas (TCGA) database (https://cancergenome.nih.gov). The binding sites between miR-145-5p and NUAK1 were predicted using TargetScan database (www.targetscan.org).

### Clinical samples

Liver cancer tissues and matched adjacent tissues (>5cm from the tumor) of 30 patients collected in outdo biotech company (Shanghai, China), and passed the review of the ethics committee of Taizhou hospital, Zhejiang province (2005DKA21300). None of these patients received preoperative chemotherapy or radiotherapy or targeted drug therapy. Collect tissue samples and freeze them in liquid nitrogen. All samples obtained informed consent and approved by the Medical Ethics Committee of Taizhou Hospital, Zhejiang Province review committee. Details of the tissue samples are shown in supplementary table.

### Cell culture

Five liver cancer cell lines were called SNU-387, SNU-449, HepG2, MHCC97H, HCCLM3. Human normal hepatocyte cell line MIHA, SNU-387, SNU-449 and HepG2 were purchased from American Type Culture Collection (Manassas, VA), HCCLM3 was purchased from liver cancer institute of Fudan University (Shanghai, China), and MHCC97H was gifted from the College of life Sciences, Jilin University. The HepG2 cell line was confirmed by STR profiling. All of them were maintained in the recommended culture condition and incubated at 37˚C in a humidified environment containing 5% CO_2_. Reverse transcription quantitative polymerase chain reaction (RT-qPCR) was performed in order to select the cell lines with the highest HOTAIR, NUAK1 and lower miR-145-5p expression for further experimentation.

### Cell transfection

Liver cancer cells were inoculated in culture dishes 24h prior to transfection. For enhance or decreased expression of HOTAIR and miR-145-5p. Short hairpin RNA (shRNA) (5'-GAACGGGAGTACAGAGAGA-3') sequences targeting human HOTAIR and a non-target sequence (sh-NC) (5'-TTCTCCGAACGTGTCACGT-3') were constructed by GenePharma (Shanghai, China). A full-length human HOTAIR expression vector was purchased from Addgene (LZRS-HOTAIR, #26110). The negative control for LZRS-HOTAIR is LZRS vector. Si-NC (sence 5'-UUCUCCGAACGUGUCACGUTT-3', antisense 5'-ACGUGACACGUUCGGAGAATT-3'), Si-EZH2 (sense 5'-GCUAAGGCAGCUGUUUCAGTT-3', antisense 5'-CUGAAACAGCUGCCUUAGCTT-3'), miR-145 mimics (sence 5'-GUCCAGUUUUCCCAGGAAUCCCU-3', antisence 5'-CAGGUCAAAAGGGUCCUUAGGGA-3'), miRNA mimics NC (sence 5'-UUUGUACUACACAAAAGUACUG-3', antisense 5'-AAACAUGAUGUGUUUUCAUGAC-3'), miR-145 inhibitor (5'-AGGGAUUCCUGGGAAAACUGGAC-3'), and miRNA inhibitor NC (5'-CAGUACUUUUGUGUAGUACAAA-3') were synthesized from Ribobio (Guangzhou, China). NUAK1 inhibitor (HTH-01-015) was purchased from MCE (Shanghai, China). The vectors and microRNAs were transfected into SNU-387 and HepG2 cells using lipofectamine 3000 reagents following the instructions (invitrogen, USA).

### qRT-PCR

The total RNA from liver cancer tissues and cells was extracted by TRIzol ®reagent (Invitrogen, USA), and 1ug was taken from the extracted RNA and turned into cDNA using a reverse transcription kit (Bimake, China). The expression level of mRNA was detected by qRT-PCR instrument using SYBR qPCR mix (Bimake, China). All miRNAs were extracted from cells through EasyPure miRNA kit (TransGen Biotech, China), and then 5μl of extracted total RNA was taken and reverse transcribed into cDNA with TranScript miRNA First-Strand cDNA Synthesis SuperMix (TransGen Biotech, China). The endogenous gene Actin and U6 were used as the regulatory gene, and the target gene was standardized. Each sample was tested three times. The primers used are exhibited in Table 1.

### Transwell assay

Generally, 1×10^5^ liver cancer cells were placed in the 24-well chemotaxis chambers (Corning, CA, USA). The cells were cultured in serum-free medium with Matrigel (50:1; BD Biosciences) in the upper chamber. The lower chamber was cultured with 20% serum for 48 hours. The invaded cells were immobilized with methanol and stained with crystal violet.

### Wound healing assay

The trypsinized HepG2 and SNU-387 cells were plated in a 6-well plate. After 24 hours, scratch with a 10ul sterile pipette tip, then wash twice with PBS, and change to serum-free medium. After 48 hours, take a microscope image of the scratch.

### Western blot assay

The protein concentration was determined by coomassie G250 assay (Beyotime Biotechnology, Shanghai, China). Approximately 15-20ug total protein was added to each hole, and proteins of different molecular weights were separated by SDS-PAGE gel. Proteins with different molecular weights were transferred to the PVDF membrane (Milipore, USA). Then seal with 5% non-fat dry milk powder for 90min at room temperature and incubated using primary antibodies overnight at 4℃, which were NUAK1 (1:500, Proteintech, USA), E-cadherin (1:1000, Proteintech, USA), N-cadherin (1:2000, Proteintech, USA), Vimentin (1:2000, Proteintech, USA), EZH2 (1:1000, Proteintech, USA), MMP2 (1:500, Wanleibio, China), MMP9 (1:1000, Wanleibio, China). Then rabbit or mouse antibodies labeled with horseadish peroxidase were incubated and the signals were measured by enhanced chemiluminescence.

### Chromatin immunoprecipitation (ChIP)

ChIP assays were performed using a Chromatin Immunoprecipitation Assay Kit (Cell Signaling Technology, USA) according to the manufacturer's instructions. Following overnight incubations with the immunoprecipitating antibodies against normal rabbit immunoglobulin G (IgG) (Cell Signaling Technology, USA), H3K27me3 (abcam, US) and EZH2 (Proteintech, USA). ChIP-qPCR analyses was performed after this step. Immunoprecipitated DNA (2μL/reaction) was used for qPCR amplification of the pmiR-145p regulatory region using primer sets (The primer sequence of the pmiR-145p promoter region is shown in table 1). Quantification of the immunoprecipitated DNA was performed using qPCR. The ChIP data were calculated as a percentage relative to the input DNA using the equation 2: input Ct- Target Ct × 0.1 × 100.

### Luciferase reporter assay

The cells were digested and centrifuged and counted and spread in 96-well plates at a rate of 8000-10000/ well. 1μg wild type or mutant plasmid (Gene Pharma, China) and miR-145 mimic were transfected, and the medium was changed 6 h later. 48 hours later, the cell lysate was used for cracking for 30 minutes to 1 hour, and firefly luciferase reaction solution and sea kidney luciferase reaction solution were added for detection.

### Immunofluorescence

The transfected cells were inoculated on a sterile glass cover and cultured overnight. After washing with PBS, the transfected cells were fixed with 4% paraformaldehyde and permeabilized with 0.25% Triton-x. Finally, 3% Bovine albumin blocking and N-cadherin antibody immunofluorescence staining were used, Hoechst 33342 was used for nuclear staining. The cell staining results were photographed with a FV1000 confocal laser microscope (Tokyo, Japan) at 40 x.

### Statistical analysis

All data were displayed as the mean ± SD for more than three independent experiments. Data were analyzed by GraphPadPrism8 software. Unpaired student's t-test was applied to compare two groups, and one-way ANOVA was used for the comparison among multiple groups. P value was less than 0.05, which was considered to be statistically significant.

## Results

### HOTAIR was highly expressed in liver cancer tissues and cells

**Figure 1:**
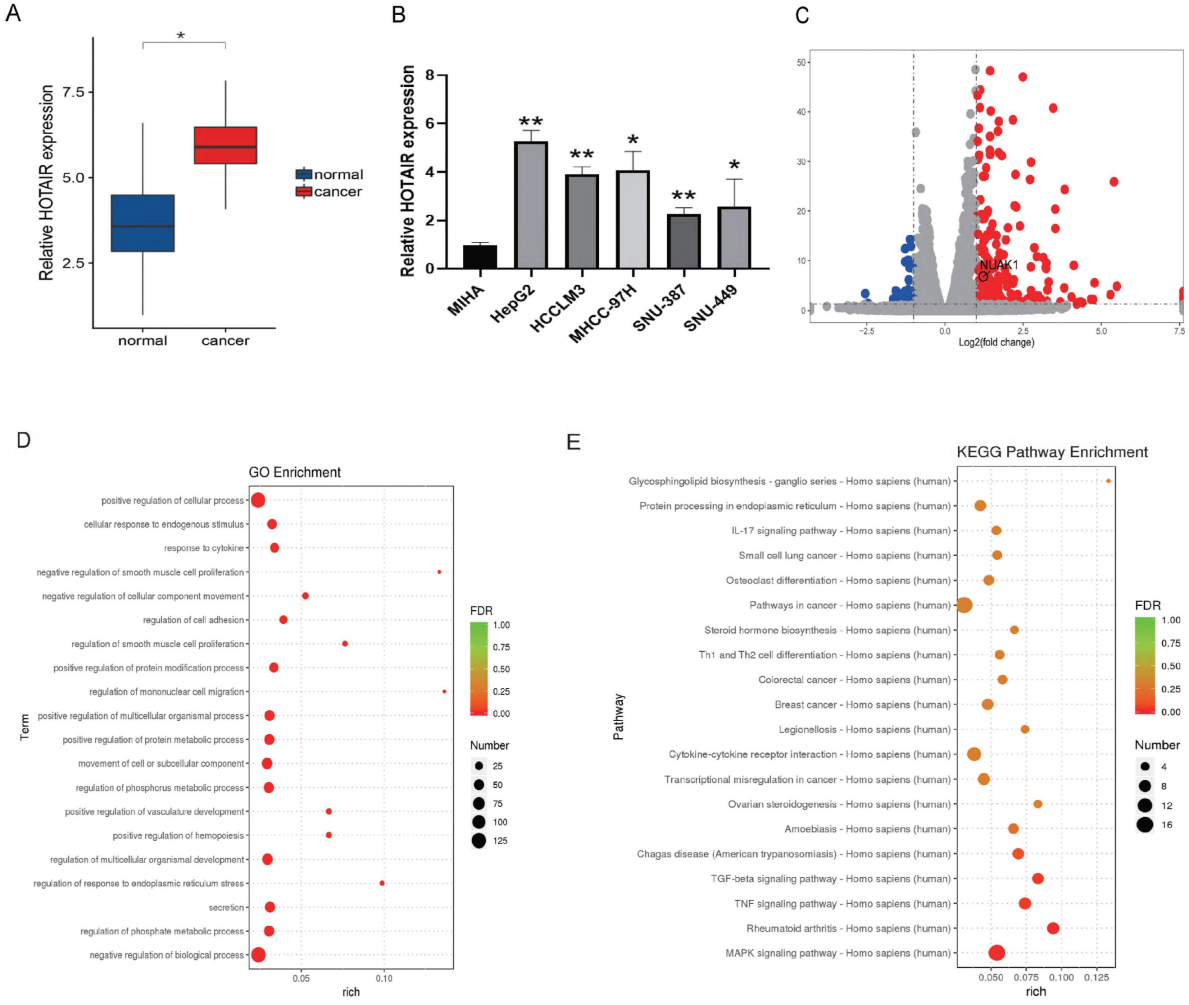
** HOTAIR is highly expressed in liver cancer tissues and cells. (A)** qPCR experiment to detect the RNA expression of HOTAIR in 30 pairs of HCC tissue samples. **(B)** Detected the expression of HOTAIR in normal liver cells and five types of liver cancer cells. **(C)** Transfected the LZRS-HOTAIR plasmid into HepG2, and detected mRNA expression changes by transcriptome sequencing analysis. **(D)** GO enrichment analyzed gene enrichment items. **(E)** KEGG analyzed gene enrichment pathways. The data shown were representative of three independent experiments. Bars, SD (n=3), *p<0.05, **p<0.01, and ***p<0.001 vs NC group.

To gain insight into the biological role of HOTAIR in human liver cancer development, we analyzed the expression of HOTAIR in 30 pairs of liver cancer tissues and adjacent normal hepatic tissues by qRT-PCR. We found that HOTAIR were more highly expressed in liver cancer tissues compared with adjacent normal tissues. (Fig. 1A). We also tested the expression of HOTAIR in five liver cancer cell lines (HepG2, HCCLM3, MHCC-97H, SNU-387, SNU-449) and normal liver cells (MIHA) and found that the expression of HOTAIR was significantly increased in liver cancer cells compared with the normal liver cell line (Fig. 1B).

We next used plasmid-mediated overexpression to increase the expression of HOTAIR in HepG2 cells and performed transcriptome sequencing to examine differentially expressed genes in these cells compared with controls. The results revealed 278 up-regulated genes and 78 down-regulated genes, and the expression of HOTAIR and NUAK1 were positively correlated (Fig. 1C). The RNA-Seq data have been deposited in the NCBI SRA with accession number PRJNA758831. GO functions include cell adhesion, invasion and metastasis (Fig. 1D). KEGG pathway analysis of the differentially expressed genes identified involvement in MAPK, TNF, and TGF-β pathways (Fig. 1E). These results indicate that the high expression of HOTAIR in cancer tissues and cells may affect the invasion and metastasis of liver cancer.

### HOTAIR affected the invasion and metastasis of liver cancer cells

**Figure 2:**
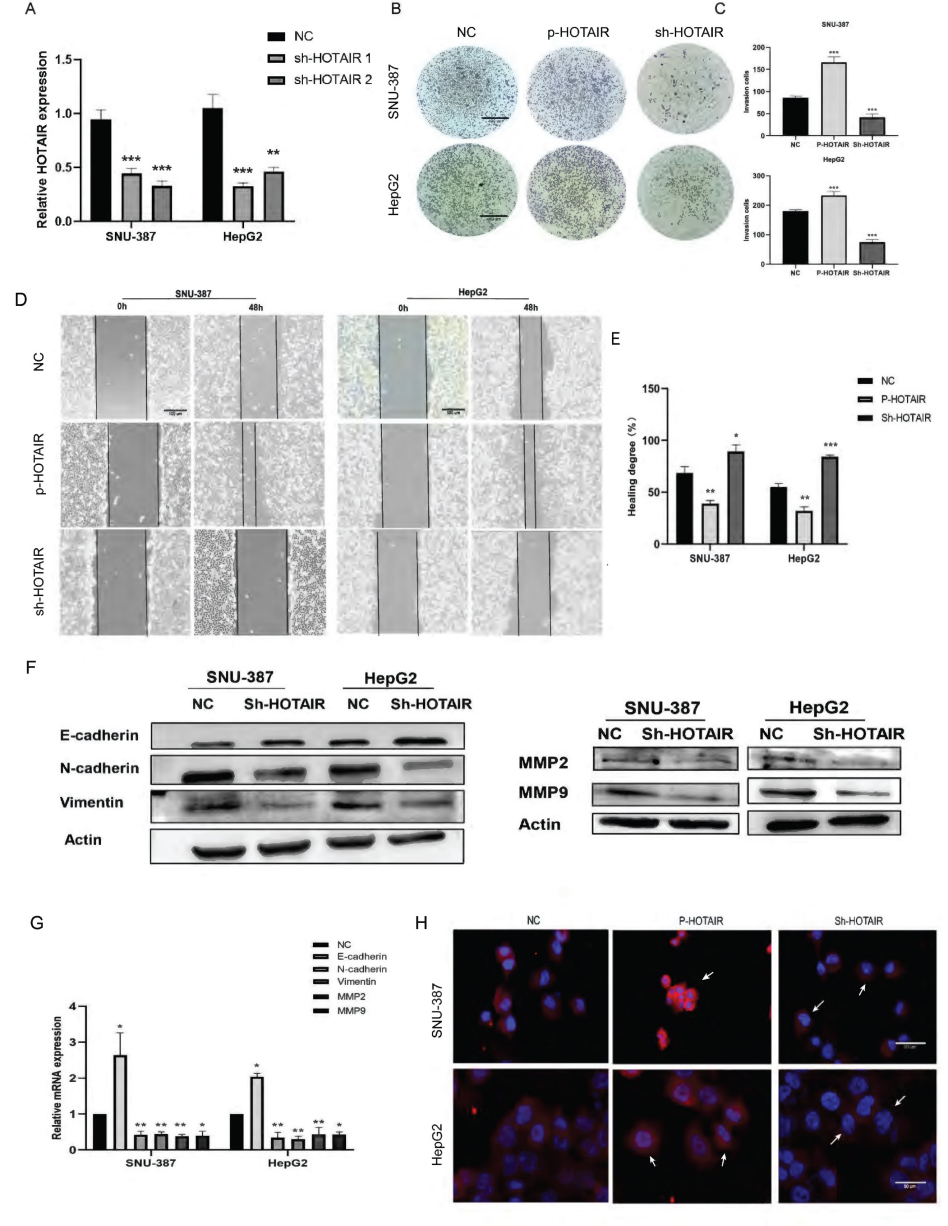
** HOTAIR affects the invasion and metastasis of liver cancer cells. (A)** Tested the transfection efficiency of Sh-HOTAIR plasmid. **(B-C)** The transwell experiment detected the invasion ability of cells. **(D-E)** Scratch test detected cell migration ability. (F) Detected the relationship between HOTAIR and EMT related proteins by Western blot. **(G)** qPCR experiment to detect the RNA expression levels of E-cadherin, N-cadherin, Vimentin, MMP2, MMP9 genes.** (H)** Detected the protein expression level of N-cadherin by immunofluorescence method. Added arrows to indicate cells of interest. The data shown were representative of three independent experiments. Bars, SD (n=3), *p<0.05, **p<0.01, and ***p<0.001 vs NC group.

We next performed *in vitro* experiments to examine the effect of HOTAIR on the invasion and metastasis of liver cancer cells. HepG2 is human hepatoblastoma and SNU-387 is human hepatocellular carcinoma cell line. We chose HepG2 and SNU-387 used for subsequent experiments because the expression of HOTAIR in both cells was high, which would be more illustrative in the subsequent Si-HOTAIR experiment. At the same time, we also conducted overexpression experiments in both cells to further illustrate the role of HOTAIR with different degrees of cancer. The transfection efficiency of overexpressed HOTAIR has been included in supplementary data (Supplementary Fig. A). Two different HOTAIR knockout plasmids (Sh-HOTAIR1, Sh-HOTAIR2) were used to reduce the expression of HOTAIR in SNU-387 and HepG2 cells, and the efficiency of HOTAIR knockdown was detected by q-PCR (Fig. 2A). We selected Sh-HOTAIR1 for experiments, as it showed better knockdown effects.

Through wound scratch and Transwell experiments, we found that the decrease of HOTAIR in liver cancer cells reduced cell migration and invasion ability (Fig. 2B, 2C, 2D and 2E). In contrast, increased expression of HOTAIR enhanced the invasion and metastasis ability of the two cell lines.

To determine whether HOTAIR can affect the expression of EMT-related gene, we examined the effect of HOTAIR knockdown on the protein expression of E-cadherin, N-cadherin, Vimentin, MMP2 and MMP9 by western blot analysis.

Compared with sh-NC group, cells with decreased HOTAIR expression showed increased expression of E-cadherin protein and decreased protein expression of N-cadherin, Vimentin, MMP2 and MMP9 (Fig. 2F). Meanwhile, we also examined the mRNA expression levels of E-cadherin, N-cadherin, Vimentin, MMP2 and MMP9 by qPCR, and the results were consistent with the protein levels (Fig. 2G). We examined the expression of N-cadherin in liver cancer cells with HOTAIR overexpression (P-HOTAIR) or knockdown (Sh-HOTAIR) by immunofluorescence and found that compared with the NC group, the Sh-HOTAIR group showed reduced N-cadherin expression, while the P-HOTAIR group showed increased N-cadherin expression (Fig. 2H). These results indicate that HOTAIR expression may affect the invasion and metastasis of liver cancer cells through regulating EMT and MMP enzymes.

### HOTAIR induced EMT through NUAK1

**Figure 3:**
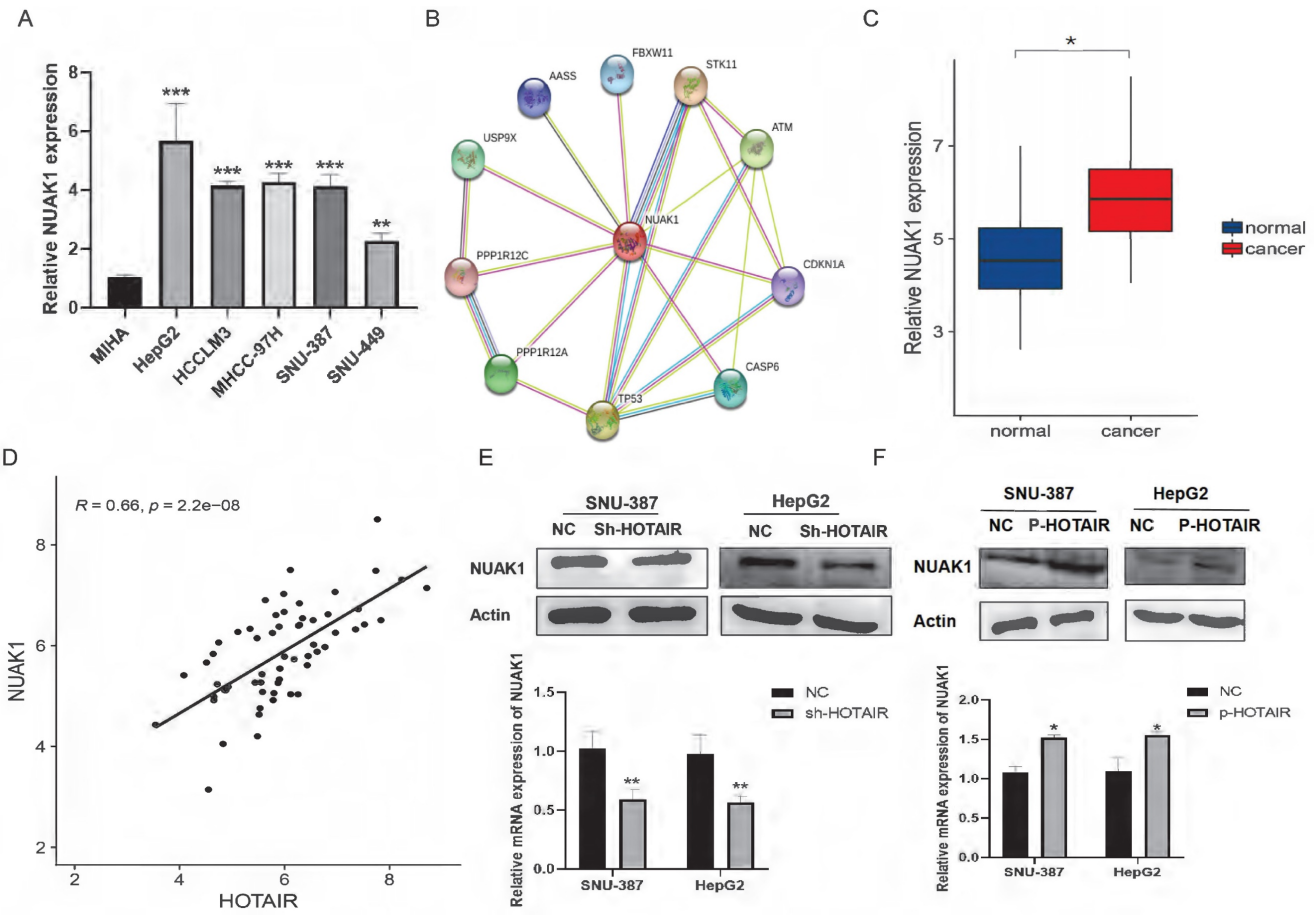
** HOTAIR and NUAK1 are positively correlated. (A)** Detected the NUAK1 expression levels in six cell lines. **(B)** Analyzed NUAK1 interacting genes through STRING website. **(C)** qPCR experiment to detect the mRNA expression of NUAK1 in HCC tissue samples.** (D)** Relative analysis of HOTAIR and NUAK1 expression in HCC tissues. **(E-F)** Detected the relationship between HOTAIR and NUAK1 by Western blot and qPCR. The data shown were representative of three independent experiments. Bars, SD (n=3), *p<0.05, **p<0.01, and ***p<0.001 vs NC group.

Transcriptome sequencing analysis results identified the NUAK1 gene among the 278 up-regulated genes in HepG2 cells. The AMPK family kinase NUAK1 is a direct target of AKT and is phosphorylated and activated by AKT. We examined the expression of NUAK1 in five liver cancer cell lines and found that NUAK1 was highly expressed in these cell lines (Fig. 3A). Analysis using the STRING database revealed that NUAK1 interacts with a variety of proteins that regulate invasion and metastasis (Fig. 3B). We also found that NUAK1 was highly expressed by qPCR in 30 pairs of liver cancer tissues compared with adjacent normal tissues (Fig.3C) and that expressions of HOTAIR and NUAK1 were positively correlated (Fig. 1A, 3C, and 3D).

To validate the transcriptome sequencing results, we performed western blot and qPCR in liver cancer cell lines. The results showed that down-regulated expression of HOTAIR significantly decreased the protein and mRNA expression of NUAK1 in SNU-387 and HepG2 cells (Fig. 3E). Overexpression of HOTAIR has the opposite result (Fig. 3F). These data suggest that the expression of HOTAIR is positively correlated with the expression of NUAK1.

**Figure 4:**
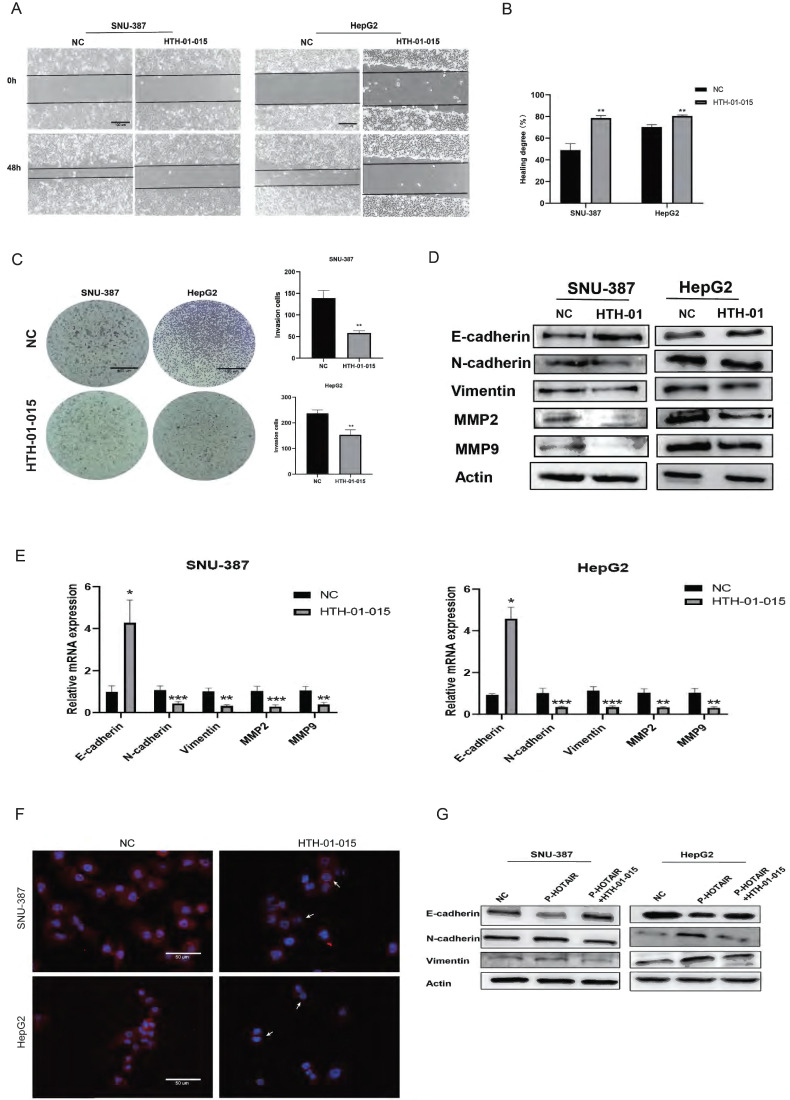
**HOTAIR regulates EMT through NUAK1 (A-B)** Scratch test detected cell migration ability. **(C)** Added HTH-01-015 to SNU-387 and HepG2 cells, and Transwell test to detect cell invasion ability. **(D)** Added HTH-01-015 to SNU-387 and HepG2 cells, and detected the protein expression of E-cadherin, N-cadherin, Vimentin, MMP2, MMP9 by Western blot. **(E)** Added HTH-01-015 to SNU-387 and HepG2 cells, and detected the mRNA expression of E-cadherin, N-cadherin, Vimentin, MMP2, MMP9 by qPCR.** (F)** Immunofluorescence test to detect the protein expression level of N-cadherin. Added arrows to indicate cells of interest. **(G)** Transfected LZRS-HOTAIR plasmid in SNU-387 and HepG2 cells, and co-transfected LZRS-HOTAIR plasmid and HTH-01-015, used Western blot to detect the protein expression of E-cadherin, N-cadherin, Vimentin. The data shown were representative of three independent experiments. Bars, SD (n=3), *p<0.05, **p<0.01, and ***p<0.001 vs NC group.

To evaluate the role of NUAK1 in liver cancer invasion and migration, we next used HTH-01-015, an inhibitor of NUAK1, in transwell and scratch assays in SNU-387 and HepG2 cells. The results showed that inhibition of NUAK1 reduced the invasion and migration ability of liver cancer cells (Fig. 4A, 4B, and 4C). Western blot and qPCR analysis of EMT markers revealed that the protein and mRNA expression of E-cadherin increased in NUAK1 inhibited cells compared with control cells, while the protein and mRNA expressions of N-cadherin, Vimentin, MMP2 and MMP9 decreased (Fig. 4D and 4E). Immunofluorescence of N-cadherin in cells treated with HTH-01-015 showed that the expression of N-cadherin protein was reduced compared with control cells (Fig. 4F). Together these results indicate that NUAK1 is highly expressed in liver cancer tissues and cells and may affect invasion and metastasis by inducing EMT.

To examine whether HOTAIR regulates EMT in liver cancer cells through NUAK1, we transfected cells with the HOTAIR overexpression plasmid (P-HOTAIR) followed by addition of the NUAK1 inhibitor HTH-01-015 and then detected EMT markers E-cadherin, N-cadherin, and Vimentin by western blot. We found that addition of the NUAK1 inhibitor reversed the effect of HOTAIR overexpression on EMT marker levels (Fig. 4G). These results indicate that HOTAIR induces EMT through NUAK1 in liver cancer cells.

### miR-145-5p affected invasion and metastasis by regulating NUAK1

**Figure 5:**
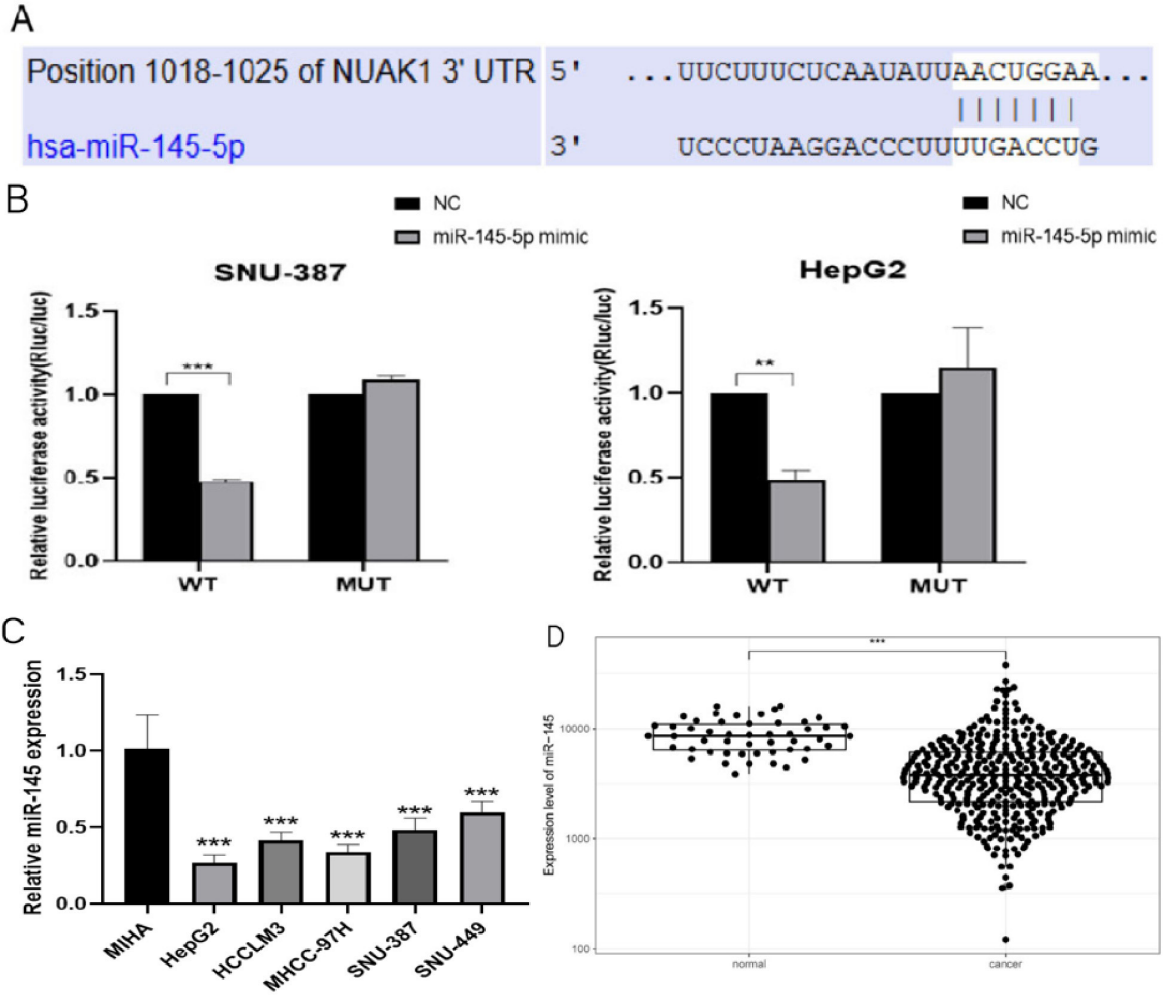
** miR-145-5p is low expressed in liver cancer (A)** The target of NUAK1 predicted by TargetScan Human 7.2. **(B)** Firefly luciferase activity normalized to that of Renilla luciferase 48 h after transfection of SNU-387 and HepG2 cells with reporter vectors expressing wild-type or mutant NUAK1 3'UTR. **(C)** Detected miR-145-5p expression in five liver cancer cells by qPCR. **(D)** Analyzed the expression of miR-145-5p in HCC tissues by TCGA database. The data shown were representative of three independent experiments. Bars, SD (n=3), *p<0.05, **p<0.01, and ***p<0.001 vs NC group.

We next used TargetScan (www.targetscan.org) to examine potential upstream regulatory factors of NUAK1 and predicted miR-145-5p is a candidate regulator of NUAK1 (Fig. 5A). We demonstrated that miR-145-5p does indeed bind to the 3' untranslated region of NUAK1 mRNA and thus directly regulates NUAK1 by dual luciferase reporter method (Fig. 5B). We analyzed the expression of miR-145-5p in five liver cancer cell lines and found that the expression of miR-145-5p was significantly decreased compared with the normal liver cell line (Fig. 5C). We also tested the expression of miR-145-5p in liver cancer tissues through the TCGA database and found that the expression of miR-145-5p was significantly lower in liver cancer tissues compared with adjacent tissues (Fig. 5D).

**Figure 6:**
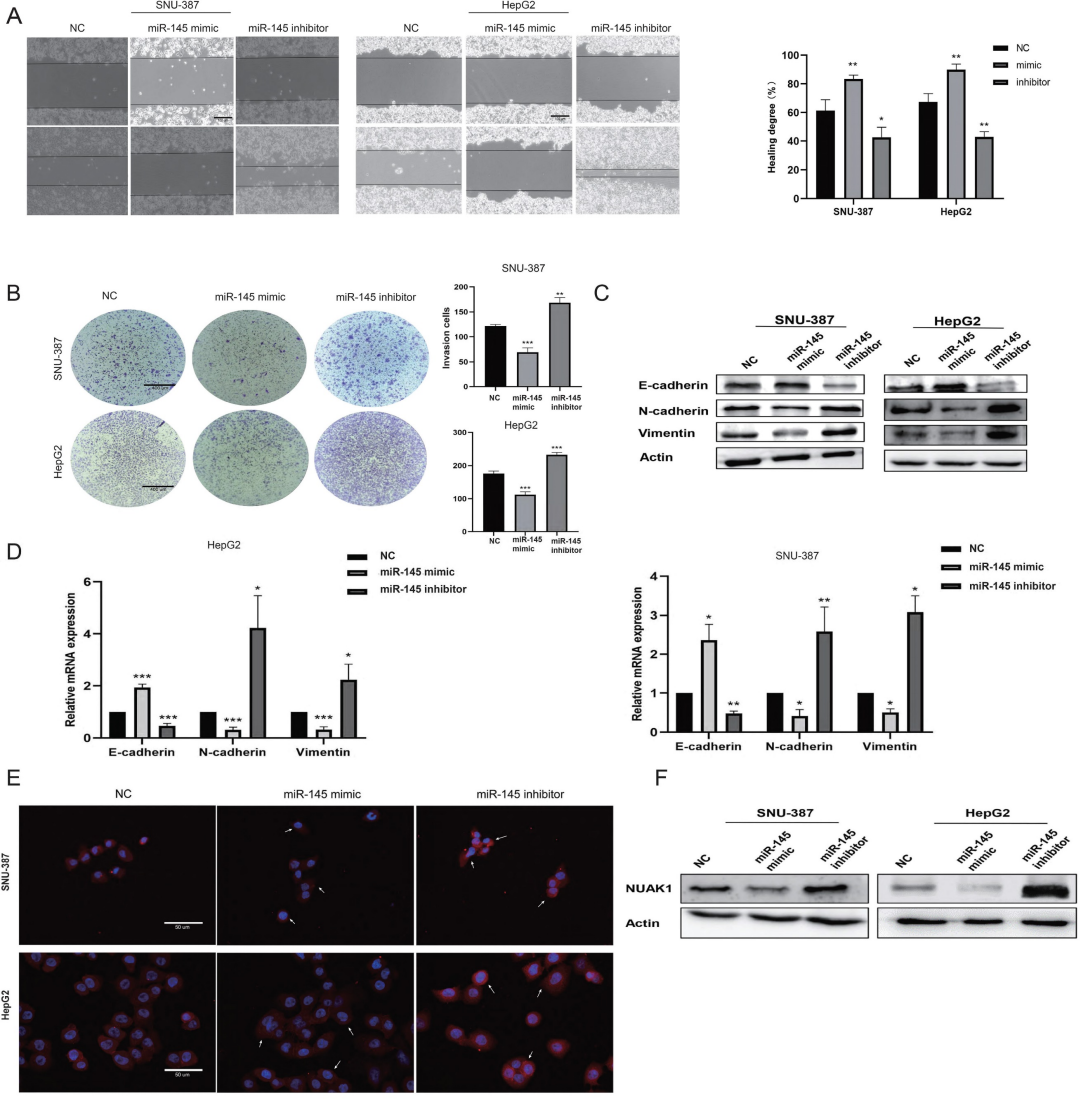
**miR-145-5p is the upstream target gene of NUAK1. (A)** Scratch test detected cell migration ability. **(B)** Transfected miR-145 mimic and inhibitor in SNU-387 and HepG2, Transwell test to detect cell invasion ability. **(C)** Transfected with miR-145 mimic and inhibitor, Western blot to detect the protein expression of E-cadherin, N-cadherin, and Vimentin. **(D)** qPCR experiment to detect mRNA expression of E-cadherin, N-cadherin, Vimentin, MMP2, MMP9. **(E)** Immunofluorescence test to detect the protein expression level of N-cadherin. Added arrows to indicate cells of interest.** (F)** In cells transfected with miR-145 mimic and inhibitor, Western blot was used to detect the protein expression of NUAK1. The data shown were representative of three independent experiments. Bars, SD (n=3), *p<0.05, **p<0.01, and ***p<0.001 vs NC group.

We next evaluated the effect of miR-145-5p on cell invasion and metastasis by scratch and transwell experiments. The transfection efficiency of miR-145-5p was detected by qPCR (Supplementary Fig. B). The invasion and metastasis abilities of miR-145 mimic-transfected cells were reduced compared with NC cells, while the invasion and metastasis abilities of cells transfected with miR-145 inhibitor were enhanced (Fig. 6A, 6B). Western blot analysis showed that the expression of E-cadherin was increased in cells transfected with miR-145 mimic, while the expressions of N-cadherin and Vimentin were decreased. Transfection with the miR-145 inhibitor showed the opposite results (Fig. 6C). qPCR assay results were consistent with the protein expression patterns (Fig. 6D). Immunofluorescence showed that the expression of N-cadherin in the miR-145 mimic group was reduced compared with NC group, while N-cadherin expression was increased in cells transfected with miR-145 inhibitor group (Fig. 6E).

To explore whether miR-145-5p regulates the expression of NUAK1 in liver cancer cells, we next transfected miR-145 mimic and miR-145 inhibitor in SNU-387 and HepG2 cells and examined NUAK1 expression by western blot analysis. We found that the expression of NUAK1 in the miR-145 mimic group was reduced compared with NC, while NUAK1 protein expression in the miR-145 inhibitor group was increased (Fig. 6F). These results indicate that miR-145 may affect the invasion and metastasis of liver cancer cells by regulating the expression of NUAK1 and regulating the EMT process.

### HOTAIR silenced miR-145-5p expression by recruiting PRC2

**Figure 7:**
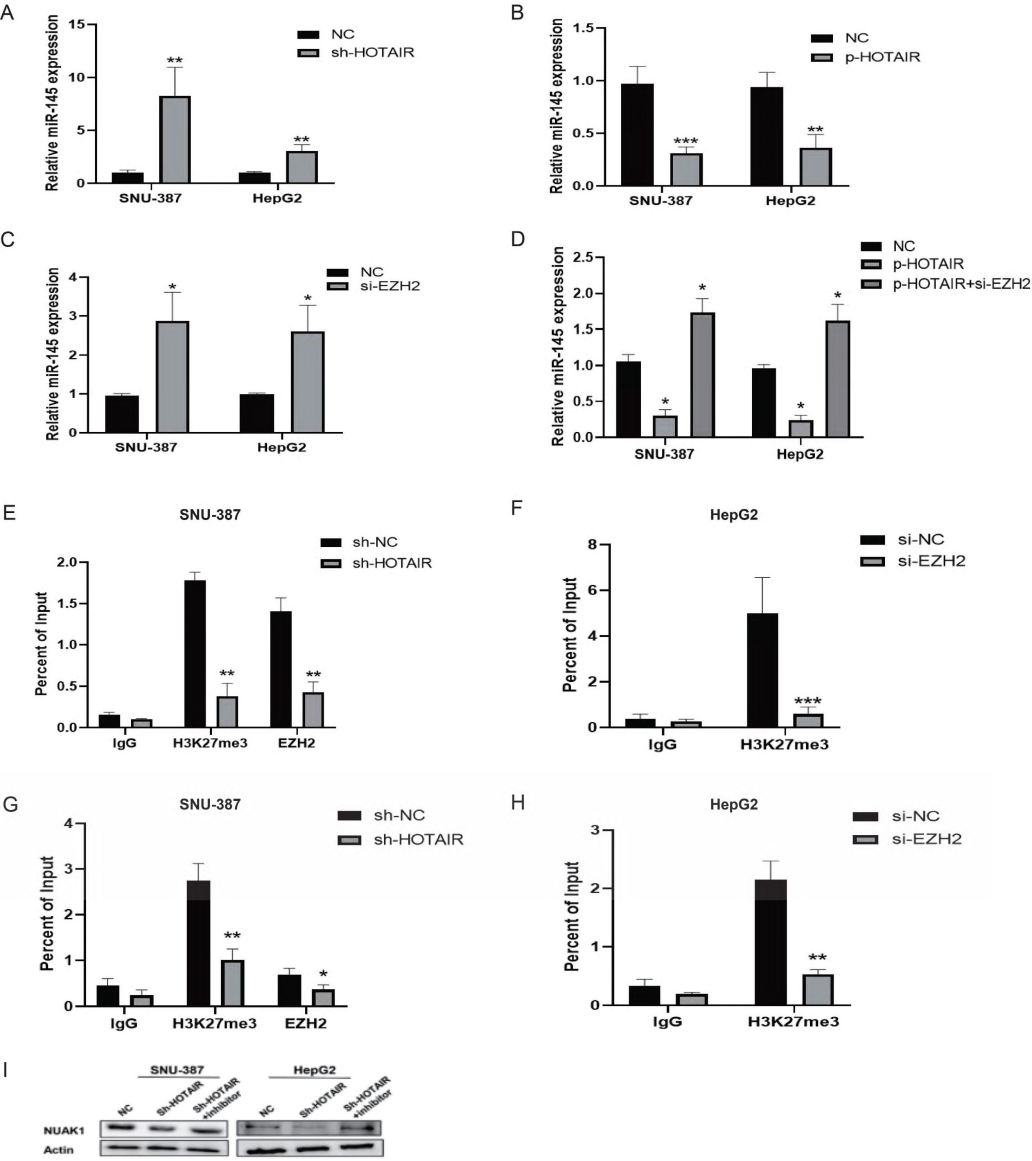
** HOTAIR regulates the expression of NUAK1 through miR-145-5p. (A-B)** Transfected Sh-HOTAIR and LZRS-HOTAIR plasmids in SNU-387 and HepG2, and detected the expression level of miR-145-5p by qPCR. **(C)** Transfected si-EZH2 in SNU-387 and HepG2, and detected the expression level of miR-145-5p by qPCR. **(D)**Transfected LZRS-HOTAIR plasmid in SNU-387 and HepG2, and transfected LZRS-HOTAIR plasmid while adding si-EZH2, and detected the expression level of miR-145-5p by qPCR. **(E-H)** ChIP assays in SNU-387 and HepG2 transfected with sh-HOTAIR and si-EZH2 cells were performed on the miR-145-5p promoter regions using anti-H3K27me3 and EZH2 antibodies. Enrichment was determined relative to the input controls. **(I)**Transfected Sh-HOTAIR plasmid in SNU-387 and HepG2, and transfected Sh-HOTAIR plasmid while adding miR-145 inhibitor, Western blot experiment to detect the protein expression of NUAK1. The data shown were representative of three independent experiments. Bars, SD (n=3), *p<0.05, **p<0.01, and ***p<0.001 vs NC group.

It is reported that 20% of the lncRNAs can bind the polycomb group protein (PcG) complex to regulate downstream gene transcription. EZH2, a key subunit of PRC2, which also includes SUZ12 and EED is a histone methyltransferase and represses downstream gene transcription by trimethylating histone H3 lysine 27 (H3K27me3). qPCR showed that the gene expression of miR-145-5p was increased in SNU-387 and HepG2 cells with low HOTAIR expression and reduced in cells with high HOTAIR expression (Fig. 7A and 7B). To determine whether HOTAIR regulates miR-145-5p expression levels by binding with PRC2, we added si-EZH2 to liver cancer cells and found that the expression of miR-145-5p increased as detected by qPCR (Fig. 7C). Meanwhile, the transfection efficiency was detected by Western blot (Supplementary Fig. C). However, upon treatment of si-EZH2 to cells with HOTAIR overexpression, EZH2 inhibition reversed the effect of HOTAIR on miR-145-5p gene expression (Fig. 7D). The results of ChIP assays showed that EZH2 could directly bind to the promoter region of miR-145-5p and mediate H3K27me3 modification, while knockdown of HOTAIR and EZH2 led to reduced EZH2 and H3K27 binding ability (Fig.7E, 7F, 7G and 7H). In conclusion, these data indicate that HOTAIR recruit the PRC2 complex to silence miR-145-5p via H3K27me3 modification.

### HOTAIR regulated the expression of NUAK1 through miR-145-5p

We next co-transfected SNU-387 and HepG2 cells with the HOTAIR knockdown plasmid and miR-145 inhibitor and examined the protein expression of NUAK1 by western blot. The results showed that the expression of NUAK1 indeed not change significantly compared with controls (Fig. 7I).

Together our results suggest that HOTAIR may regulate the miR-145-5p /NUAK1 axis through PRC2 to induce the EMT process and enhance the invasion and metastasis of liver cancer cells.

## Discussion

EMT activation is an important sign indicative of cell invasion and metastasis 28. Therefore, finding the key molecule that inhibits the biological process of EMT is an ideal target for preventing the invasion and metastasis of liver cancer. Our results showed that the lncRNA HOTAIR is overexpressed in liver cancer tissues and cell lines. HOTAIR regulates EMT by upregulating NUAK1. In addition, we found that miR-145-5p is involved in HOTAIR-mediated regulation of NUAK1. HOTAIR also affects the expression of miR-145-5p through PRC2 (EZH2). Therefore, our study findings reveal the involvement of the HOTAIR/PRC2/miR-145-5p/NUAK1 axis in the invasion and metastasis of liver cancer cells.

HOTAIR is a well-studied lncRNA, that is associated with a poor prognosis, recurrence, and metastasis of breast cancer, pancreatic cancer, gastric cancer, and other cancers 29-32. Previous studies showed that HOTAIR reverses EMT by inhibiting HoxD10 and reducing the expression of miR-7 in breast cancer 33. Hande Topel provides the first evidence suggesting a role for lncRNA HOTAIR in the modulation of c-Met to promote hybrid E/M phenotype 34. HOTAIR also contributes to cell progression in HCC by sponging miR-217-5p 35. In liver cancer stem cells, HOTAIR reduces H3k36me3 by inhibiting the expression of SETD2 and promotes the malignant growth of human liver cancer stem cells 36. IKKα, IKKβ, and IKKγ promote or inhibit the malignant progression of HCC stem cells by altering telomere length and relying on HOTAIR-dependent telomerase activity 37. Consistent with the published data, we confirmed that changes in HOTAIR expression have an impact on the invasion and metastasis of liver cancer cells. Western blot verified that changes in HOTAIR expression affected the expression of EMT marker proteins E-cadherin, N-cadherin and Vimentin. The above results indicate that HOTAIR regulates the invasion and metastasis of liver cancer cells by regulating the expressions of EMT-related factors.

NUAK1 a protein kinase of the AMPK family is identified to be involved in the regulation of a variety of genes that regulate tumor invasion and metastasis 17. Two methods of NUAK1 activation have been reported: a tumor suppressor LKB1-dependent NUAK1 activation; in cells lacking LKB1 38, modulation of NUAK1 activity through the calcium-dependent PKC pathway 39. Whether HOTAIR is involved in these mechanisms and whether it controls NUAK1 expression by regulating related transcription factors will be examined in future studies.

LINC00958 acts as a ceRNA to regulate the miR-625/NUAK1 axis, and affects the invasion and metastasis of nasopharyngeal carcinoma cells 40. In prostate adenocarcinoma, miR-30b induces EMT by inhibiting the expression of NUAK1 41. Here we demonstrated that HOTAIR and NUAK1 were positively correlated in liver cancer tissues and cell lines and that the addition of NUAK1 inhibitor reversed the effect of high HOTAIR expression on EMT-related proteins. These findings indicate that HOTAIR increases EMT by upregulating the expression of NUAK1.

We also explored the mechanism by which the lncRNA HOTAIR modulates NUAK1. Many studies have reported the correlation and regulatory mechanisms between lncRNAs and miRNAs in cancers. lncRNA can regulate miRNA expression through the PRC2 complex or lncRNA can act as competing endogenous RNAs to regulate miRNA expression 42. In gastric cancer, HOTAIR regulates the expression of miR-34a through PRC2, thereby affecting the invasion and metastasis of gastric cancer 43. HOTAIR also up-regulates DNMT through EZH2 by epigenetically inhibiting the expression of miR-122 in HCC 44. HOTAIR as a dynamic regulator of the gluteal adipocyte transcriptome and epigenome with functional importance for human regional AT development. Our study showed that miR-145-5p affected the invasion and metastasis of liver cancer cells. Overexpression of miR-145-5p inhibited the expression of NUAK1 and reversed the effect of HOTAIR overexpression on NUAK1. Using si-EZH2, we found that the expression of miR-145-5p increased in SNU-387 and HepG2 cells, and si-EZH2 reversed the effect of HOTAIR overexpression on miR-145-5p. In addition, we demonstrated that HOTAIR recruits and binds to PRC2 (EZH2) to epigenetically silence miR-145-5p expression via H3K27me3 modification to promote liver cancer cell-EMT process and metastasis.

In conclusion, here we found that the higher expression of HOTAIR and NUAK1 and the lower expression of miR-145 promote the invasion and metastasis of liver cancer cells. We demonstrated a positive regulatory relationship between HOTAIR and NUAK1 in liver cancer tissues and cells and showed that HOTAIR regulates the miR-145-5p /NUAK1 axis through PRC2 (EZH2). This pathway plays an important role and might be important targets for the control invasion and metastasis of liver cancer.

## Supplementary Material

Supplementary figure and table.

## Figures and Tables

**Table 1:** Primers

	Forward	Reverse
HOTAIR	5′-GGTAGAAAAAGCAACCACGAAGC-3′	5′-ACATAAACCTCTGTCTGTGAGTGCC-3′
E-cadherin	5′-TGCCCAGAAAATGAAAAAGG-3′	5′-GTGTATGTGGCAATGCGTTC-3′
N-cadherin	5′-GGTGGAGGAGAAGAAGACCAG‐3′	5′-GGCATCAGGCTCCACAGT-3′
Vimentin	5′-GCCCTTAAAGGAACCAATGA-3′	5′-AGCTTCAACGGCAAAGTTCT-3′
MMP2	5'-GTGAAGTATGGGAACGCCG -3'	5'--GCCGTACTTGCCATCCTTCT -3'
MMP9	5′-GACCTCAAGTGGCACCACCA-3′	5′-GTGGTACTGCACCAGGGCAA-3
NUAK1	5'-CCGCTCACTGATGTAATCGT-3'	5'-GTCATCTCTCAACCATCCTCAT-3'
Actin	5'-CTCCATCCTGGCCTCGCTGT -3'	5'-GCTGTCACCTTCACCGTTCC -3'
miR-145	AGTTTTCCCAGGAATCCCTAAA	5'GCGAGCACAGAATTAATACGAG-3'
U6	5'-CTCGCTTCGGCAGCACA-3'	5'-AACGCTTCACGAATTTGCGT-3'
pmiR-145p	5'-GCTCAGATGCAGCTTCAGAA-3'	5'-CCGGAGCCAAGGTTAGAAGT-3'

## Abbreviations

HOTAIR: Hox transcript antisense RNA
NUAK1: NUAK Family Kinase 1
miR-14: microRNA-145
EMT: Epithelial-to-mesenchymal transition
MMPs: matrix metalloproteinases
ECM: extracellular matrix
MMP: mitochondrial membrane potential
EZH2: Enhancer of zeste homolog 2
PRC2: polycomb-group proteins 2
