# Supplementary material for: LncRNA HOTAIR Enhances Epithelial-to-mesenchymal Transition to Promote the Migration and Invasion of Liver Cancer by Regulating NUAK1 via Epigenetic Inhibition miR-145-5p Expression
Source: J Cancer. 2023 Jul 24;14(12):2329–43. doi: 10.7150/jca.85335 (PMC10414040; doi:10.7150/jca.85335)
Supplement: Supplementary file 1 — Supplementary figure and table. [file jcav14p2329s1.pdf]

Sample number	Gender	Age(year)	Disease Subtypes	Edmondson Grade	Tumor Size(cm)	Pathomorphological Classification	Vascular Invasion	HBsAg	Cirrhosis
1	F	50	+	I	10×6×5	Nodular	−	−	−
2	M	56	+	II	3×3×4.5	Multinodular	−	−	−
3	M	54	+	II	5×6×5.5	Nodular	−	−	−
4	M	61	+	II	3×5×2	Nodular	−	+	+
5	M	41	+	II	2×3×3	Infiltrating	+	−	−
6	F	53	+	II	4×4×5	Multinodular	−	+	+
7	M	23	+	II	9×4.5×6	Nodular	+	−	−
8	M	70	+	II	6×5×7	Infiltrating	+	−	−
9	F	62	+	II	7×4.5×6	Multiple Fusion	−	−	−
10	M	42	+	II	5×4×4	Infiltrating	+	−	−
11	M	64	+	II-III	2×1.5×3	Nodular	+	+	+
12	M	62	+	II-III	4×3×3	Infiltrating	−	−	−
13	M	42	+	II-III	13.2×10×7	Infiltrating	+	+	+
14	F	64	+	II-III	8.6×7×5	Multinodular	+	+	+
15	F	62	+	II-III	5×4×4	Infiltrating	−	+	+
16	F	47	+	II-III	5×4×4.5	Thin Beam Type	+	+	+
17	M	51	+	II-III	7×5×6.5	Nodular	+	−	−
18	M	59	+	II-III	7×7×6	Multinodular	−	+	+
19	F	41	+	II-III	7×5×5.5	Multiple Fusion	+	+	+
20	M	68	+	II-III	4.5×4.5×4	Infiltrating	−	−	−
21	F	62	+	II-III	4×3×3	Thin Beam Type	+	−	−
22	F	53	+	II-III	10×8×7.5	Nodular	−	+	+
23	M	69	+	II-III	7×5.5×6	Multinodular	+	−	−
24	F	59	+	II-III	6×5.5×7	Thin Beam Type	+	−	−
25	F	60	+	II-III	6×4.5×5.5	Nodular	+	+	+
26	M	53	+	II-III	2.5×4.5×3	Infiltrating	−	−	−
27	F	65	+	II-III	12×13×6.5	Nodular	−	+	+
28	F	55	+	III	5.5×3×3	Infiltrating	−	−	−
29	M	66	+	III	7.1×5×6	Multiple Fusion	−	+	+
30	F	73	+	IV	8×5.5×6	Nodular	+	−	−

Supplementary table

M, male; F, female; Disease Subtypes: All subtypes in this item are HCC. In order to make the table more concise, we use "+" to represent HCC; HBsAg**, hepatitis B surface antigen.**

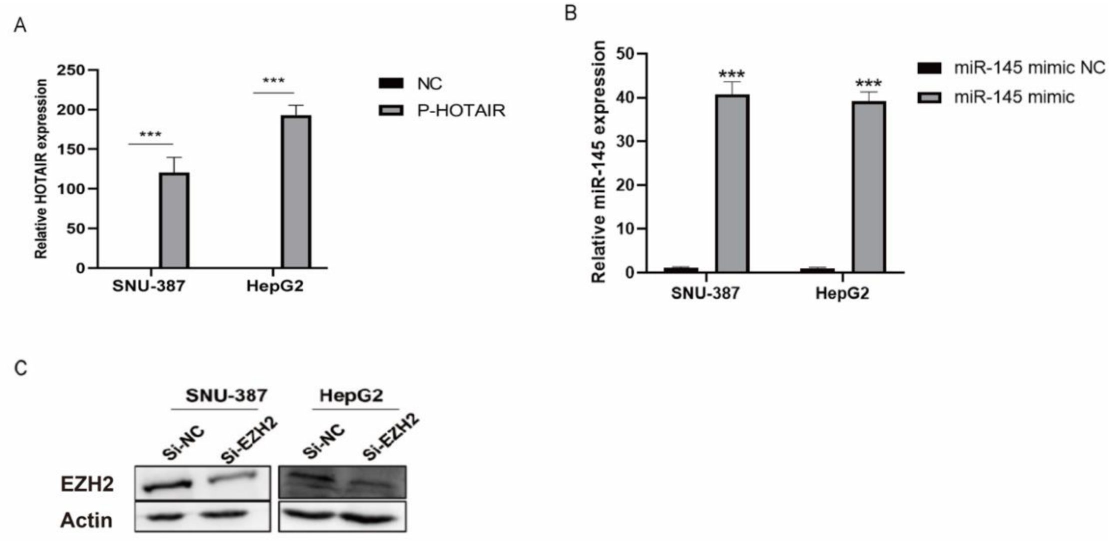

Supplementary Figure

(A) Transfected LZRS-HOTAIR plasmids in SNU-387 and HepG2, and detected the expression level of HOTAIR by qPCR. (B) Transfected miR-145 mimic in SNU-387 and HepG2, and detected the expression level of miR-145-5p by qPCR. (C) Transfected Si-EZH2 in SNU-387 and HepG2, and detected the protein expression level of EZH2 by Western blot. Bars, SD (n=3), *p<0.05, **p<0.01, and ***p<0.001 vs NC group.
